# A Systematic Review of the Characteristics and Efficacy of Recovery Training for Mental Health Staff: Implications for Supported Accommodation Services

**DOI:** 10.3389/fpsyt.2021.624081

**Published:** 2021-05-14

**Authors:** Peter McPherson, Brynmor Lloyd-Evans, Christian Dalton-Locke, Helen Killaspy

**Affiliations:** Division of Psychiatry, UCL, London, United Kingdom

**Keywords:** supported accommodation, supported housing, recovery, rehabilitation, staff development, training, systematic review

## Abstract

Evidence suggests a link between recovery-oriented practise and service user outcomes in supported accommodation settings. Current clinical guidelines recommend recovery training for supported accommodation staff, however evidence relating to the effectiveness of this type of training is unclear. This review aimed to describe and compare the characteristics and efficacy of existing recovery training packages for mental health staff. The appropriateness and applicability of the interventions was considered in relation to UK supported accommodation services. Initial search processes returned 830 papers. After duplicate removal, inclusion and exclusion criteria were applied to 489 papers, leaving a final sample of seven papers. Data were reviewed using a narrative synthesis approach. The reviewed papers showed variation in the aims, frequency, and duration of the training interventions, although all included content consistent with the five-domains of the CHIME model. All interventions used direct, in-person teaching, and prioritised interactive, experiential learning, however a number were limited by the absence of feedback, the use of one-off, rather than repeated/follow-up sessions, and a reliance on classroom-based, rather than *in-vivo*, training. There was limited evidence to suggest a consistent effect of training on staff or service user outcomes, and there was no clear association between the delivery and design characteristics of the interventions and reported outcomes. In considering the development of recovery training for supported accommodation staff, little guidance can be taken from the reviewed literature. Any training package must be developed with consideration of the unique contextual and organisational characteristics of these services. The authors recommend viewing training as one component of a broader goal of service transformation.

## Introduction

The concept of personal recovery has been established as a central policy focus for mental health services in developed countries. In line with this goal, significant investment has been made to transform services; recovery-orientation, at the service level, seeks to maximise patient autonomy and empowerment, facilitate social and community integration, and build genuine collaborative relationships between providers and service users (1). Structural factors, such as organisational culture, budgets, and leadership, have been shown to influence the recovery orientation of services (2), however research suggests ongoing confusion amongst frontline staff regarding what recovery is and how it should be facilitated (3). Staff training has been shown to influence recovery-related attitudes and knowledge (4), however the impact on service users outcomes is less clear (5).

Operating as community-based services, and staffed by non-clinical workers, mental health supported accommodation services are uniquely placed to support service users' recovery. Although definitions vary, the term supported accommodation typically describes three distinct service types: *Residential care:* time-unlimited, accommodation-based support to service users with high needs, with 24-h staffing and communal facilities; *Supported housing:* tenancies in shared or individual self-contained apartments, with staff based onsite up to 24-h per day; and, *Floating outreach* services: time-limited, visiting support to higher-functioning service users, living in self-contained, individual tenancies. Using the Simple Taxonomy for Supported Accommodation (STAX-SA), Residential care represents a Type 1 service, Supported housing a Type 2 or 3 service, and Floating outreach a Type 4 service (6). While, in the UK, recovery training for supported accommodation staff does exist, it is typically delivered in-house, using non-standardised, non-evidence-based materials. With emerging evidence suggesting a link between recovery-oriented practise within these settings and positive service user outcomes, such as successful progress to more independent (less supported) accommodation (7), interventions designed to support recovery-oriented practise must be considered a priority. Training for staff is regularly proposed as potential method of achieving this aim. Recent policy statements advocate for an urgent investment in staff training in these settings (8), and newly published NICE guidelines (9) recommend that training emphasise recovery principles, ensuring that supported accommodation staff work with a recovery-orientated approach. These positions are mirrored by researchers; as stated by Brunt et al. (10) “*Staff training, with a focus on recovery… is needed to improve the quality of care in these housing facilities*” (p. 705). It is essential, however, that, prior to the development (or adaptation) of any formalised recovery training packages for use in into theses settings, the available evidence is assessed.

A number of systematic reviews have evaluated recovery-focused interventions for mental health service users [e.g., (11)] and the nature of recovery-orientated practise [e.g., (12)], however, only recently have authors attempted to the synthesise the evidence under-pinning programmes that aim to support staff to work in a recovery-oriented manner. Jackson-Blott et al. (13) conducted a systematic review of quantitative studies (including uncontrolled and non-randomised studies) examining the effects of recovery-oriented training programs for mental health professionals; the review identified eligible 17 studies, and analysed findings using a narrative synthesis methodology. The authors highlighted methodological weaknesses of the studies (e.g., pre-post designs, limited follow-up), and variation in training characteristics across the programs, which limited their ability to draw firm conclusions regarding efficacy. The data indicated that recovery-oriented training has the potential to improve staff knowledge, attitudes and skills, in the immediate term, however there was little evidence to support longer-term maintenance, and, notably, limited evidence demonstrating an effect on service user outcomes.

The current review will attempt to extend and expand upon these findings in a number of ways. First, it will include only randomised controlled studies, thus minimising the effects of error and bias, providing a clearer indication of the efficacy of recovery-oriented training packages. Second, the review will evaluate the design and delivery characteristics of interventions from included studies against established evidence-based training methods. Finally, the review will consider the synthesised findings in relation to UK supported accommodation services, thus addressing calls for the identification/adoption of evidence-based training approaches within these settings. Specifically, this review seeks to address the following research question/s: “What is the effect of staff recovery training on service user outcomes? Is there a relationship between program characteristics and efficacy?” To address these questions, the review will: (1). Describe the content, structure and delivery methods of evaluated recovery training programs; (2). Synthesise the available evidence, in relation to service user self-reported recovery and staff recovery knowledge and behaviours; (3). Examine the relationship between training program characteristics and efficacy, and; (4). Consider these findings in relation to UK supported accommodation services.

## Materials and Methods

This review was prospectively registered with Prospero (CRD42019133559). There were no major changes between the registered protocol and completed review.

### Inclusion and Exclusion Criteria

#### Population

Any study utilising a sample of mental health staff, working in either inpatient or community settings with individuals with severe mental illness, were included. Samples could include either clinical staff (involved in the direct provision of diagnoses and/or treatment; e.g., nurses, clinical psychologists, and psychiatrists) or non-clinical staff (support service users, but do not provide diagnoses or treatment; e.g., support workers and healthcare assistants). Training interventions targeting mental health service users, students, carers, or primary care staff were excluded.

#### Recovery Training (Intervention)

Descriptions of personal recovery typically rely on the following definition by Anthony (14): “*Recovery is a deeply personal, unique process of changing one's attitudes, values, feelings, goals, skills, and/or roles. It is a way of living a satisfying, hopeful, and contributing life even within the limitations caused by illness”* (p. 527). Due the idiosyncratic nature of personal recovery, operationalising the definition has been challenging; recovery-based practise typically refers to staff practise that supports the personal recovery of service users. To avoid the definitional issues that are prevalent in this field, only training interventions that were explicitly described as “recovery” training, and aimed to effect change in staff recovery knowledge, orientation or practise, were included.

#### Comparator

No comparator-focused exclusion criteria were used.

#### Outcomes

As the purpose of staff recovery training is to improve facilitate and support personal recovery, service user, self-reported recovery was selected as the primary outcome of interest. The review also considered the following secondary outcomes: staff recovery knowledge, staff recovery practise, service user symptoms and service user functioning.

#### Study Type

The current review included randomised controlled trials, including cluster-randomised and stepped-wedge trials, assessing the efficacy of recovery training programs for mental health staff, published in English. Only trials published after 1990 were included, as personal recovery, as a concept, was only formalised after this date. No country-based limitations were imposed.

### Search Strategy

An electronic database search was conducted in April 2019. A search strategy was designed according to the PICOS structure of the review. MeSH/thesaurus terms, such as “*Mental Illness*,” “*Staff* ,” (P) “*Training*,” “*Education, Continuing*” (I), “*Mental Health Recovery*” (O), “*Trial*,” (S) were combined with free-text searches, using terms such as “*Teaching*,” “*Education*,” “*Training*,” “*Skills*,” “*Continuing professional development*.” The search strategy was applied to the following databases: MEDLINE (OVID), EMBASE (OVID) PsychInfo (OVID), CINAHL Plus (EBSCO), IBSS (ProQuest), and Cochrane Library. Limits relating to age (18+) and date (>1990) were applied. See Supplementary Files for full search strategy. Additional, potentially relevant papers were identified by reviewing reference lists of key papers.

The initial database and search results were collated, and duplicates removed; two reviewers (PM and CDL) then applied the inclusion/exclusion criteria to a random sample of the returns (10%; *n* = 49) to ensure fidelity to the screening procedure. There were no discrepancies between inclusion/exclusion decisions of the raters, indicating a reliable screening process.

### Data Extraction

A data extraction form was created, reflecting the aims of the review. The following information was recorded from each article:

- Paper characteristics: Title; Year; Journal; Country
- Study design: Population; Setting; Recruitment methods; Aim; Design; Sampling technique; Sample size/s; Sample demographics
- Experimental groups: Intervention description; Control condition;
- Recovery: Adherence to the theoretical basis of personal recovery was evaluated by examining training content with reference to the five domains of the CHIME model (15). Scores were based on a simple binary, indicating whether training content reflected each of the five domains: present vs. not present. Score (X/5).
- Training characteristics: A comprehensive, integrative review, published by Bluestone et al. (16), identified a range of in-service training design and delivery characteristics that were associated with improved learning outcomes. The findings of this study were adapted into a scorecard to provide a simple evaluation the characteristics of the included training programs: Learner engagement (passive vs. interactive); Feedback (feedback provided vs. no feedback provided); Frequency (delivered once vs. repeated delivery/follow-up sessions); Setting (classroom vs. *in-vivo*). Score (X/4).
- Outcomes: Measures; Time points; Time points reported; Results; Response rate; Unit of analysis; Statistical methods; Weighted results
- Overview: Strengths; Limitations; Conclusions.

### Quality Assessment

Quality of the included studies was assessed by the lead author (PM) using Cochrane's revised risk-of-bias tool for randomised trials [RoB2; (17)]. The tool assists the reviewer to evaluate available study information, relevant to bias, across five domains; Randomisation process; Deviations from intended interventions; Missing outcome data; Outcome measurement, and; Selection of the reported result. Based on assessments within each domain, an overall risk of bias rating is provided: “Low risk of bias,” “High risk of bias,” and “Some concerns.”

### Data Synthesis

A meta-analysis of pooled mean differences in the primary outcome was initially intended, however the low number of returns, and relative heterogeneity of included studies, in terms of intervention characteristics, made this approach unsuitable. As such, a narrative synthesis approach, informed by published guidelines (18) was used. Broadly, the method is composed of four elements: (1). Developing a theoretical model of how the interventions work, why and for whom; (2). Developing a preliminary synthesis; (3). Exploring relationships in the data, and; (4). Assessing the robustness of the synthesis product. In line with this approach, risk of bias ratings were used to support interpretation of reported data, rather than to formally weight findings or to exclude particular studies.

## Results

### Descriptives

**Figure 1:**
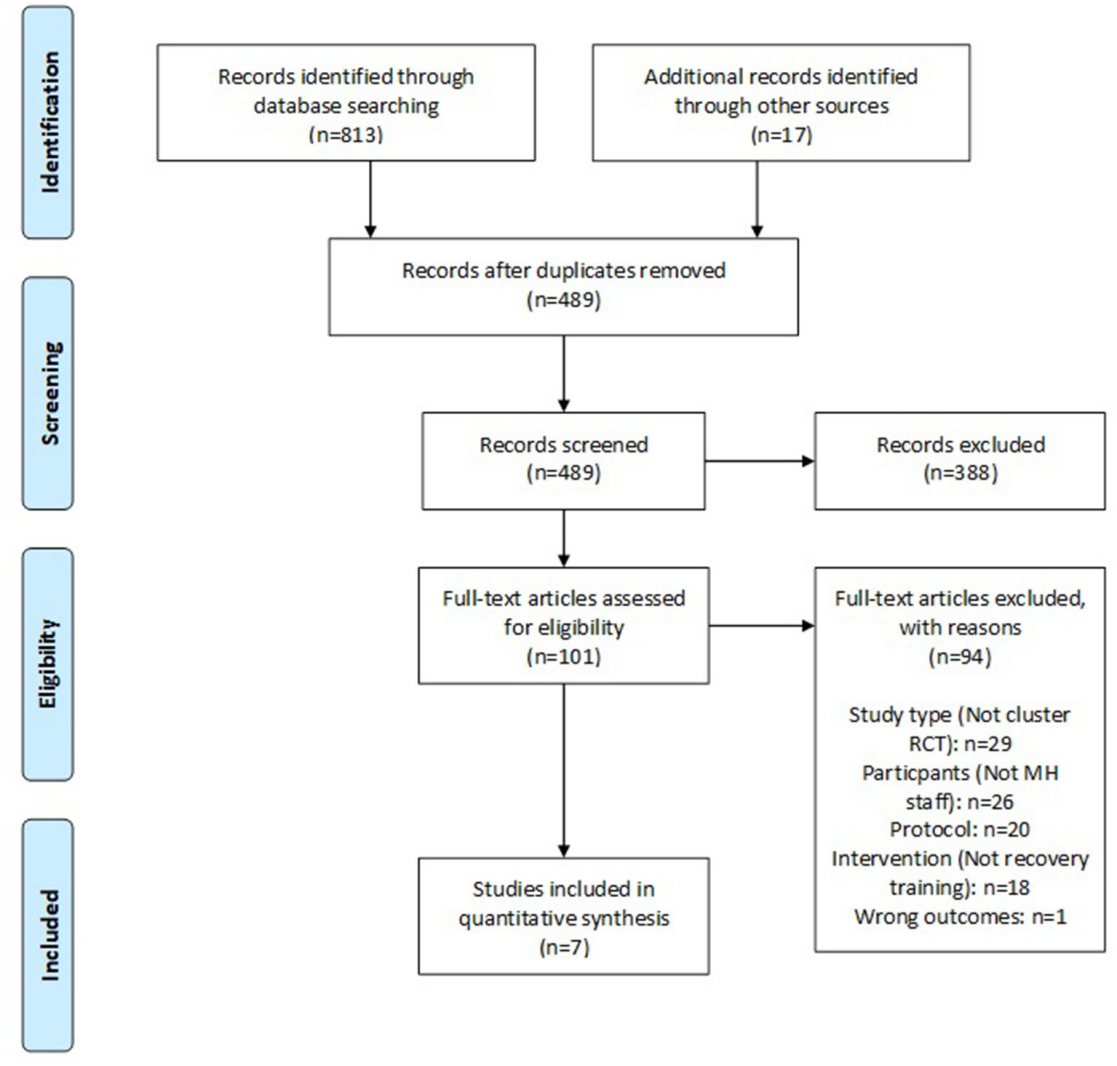
PRISMA diagram.

Initial returns comprised 813 papers from database searches and 17 from additional searches. After duplicate removal, inclusion and exclusion criteria were applied to 489 papers, leaving a final sample of seven papers. A PRISMA diagram is presented in Figure 1.

The retained papers varied broadly in terms of country, settings, service types and samples. Three were derived from work in the Netherlands [*n* = 3; (19–21)], while the remaining papers were from Hong Kong [*n* = 1; (22)], Australia [*n* = 1; (23)], Israel [*n* = 1; (24)], and England [*n* = 1; (5)]. Training programs were most commonly delivered in community (*n* = 6), rather than inpatient, settings, with samples comprised clinical staff (*n* = 3) or combined clinical and non-clinical staff (*n* = 4). No training was delivered exclusively to non-clinical staff groups. The majority of papers were based on cluster-randomised trials, with two papers based on stepped-wedge designs. Follow-up periods varied according to outcome, and ranged from simple pre-post measurement (22) to 2 year follow-up (23). Details of the included studies, including quality assessment ratings, are presented in Table 1.

**Table 1:** Details of included studies.

**Study**	**Country**	**Design**	**Setting**	**Population**	**Total sample**	Outcome/s^*^	**Assessment**	**RoB2 rating**
Bitter et al. (19)	Netherlands	Cluster RCT	Sheltered and supported housing	Social workers; nurses	14 teams/*N* = 631 SUs	SU: Recovery SU: Functioning	Mental Health Recovery Measure (MHRM; (25)) Social Functioning Scale (SFS; Birchwood et al. (26))	Low risk of bias
Bitter et al. (20)	Netherlands	Cluster RCT	Sheltered and supported housing	Social workers; nurses	14 teams/*N* = 631 SUs	Staff: Knowledge	Recovery Knowledge Inventory (RKI; Bedregal et al. (27))	Low risk of bias
Mak et al. (22)	Hong Kong	RCT	Community based services	Non-governmental “mental health service providers”	*N* = 111	Staff: Knowledge	Recovery Knowledge Inventory (RKI; Bedregal et al. (27))	Some concerns
Meadows et al. (23)	Australia	Stepped-wedge cluster RCT	Community based services	Staff from various public and community MH services	*N =* 942	SU: Recovery Staff: Knowledge	Questionnaire about the Process of Recovery (QPR; (28)) Recovery Knowledge Inventory (RKI; Meehan and Glover, (29))	Low risk of bias
Pollard et al. (24)	Israel	RCT	Inpatient units	Staff in acute and chronic inpatient units	*N =* 54	Staff: Knowledge	Practitioners' Beliefs, Goals, and Practises in Psychiatric Rehabilitation Questionnaire (PBGPPR; Casper et al. (30))	Some concerns
Slade et al. (5)	England	Cluster RCT	Community mental health teams	Multidisciplinary CMHT staff	27 teams/*N =* 403 SUs	SU: Recovery Staff: Knowledge Staff: Practise SU: Symptoms SU: Functioning	Questionnaire about the Process of Recovery (QPR; (28)) Recovery Knowledge Inventory (RKI; Meehan and Glover, (29)) Recovery Practise Scale Brief Psychiatric Rating Scale (BPRS; Overall and Gorham, (31)) Global Assessment of Functioning Scale (GAF; APA, 2013)	Low risk of bias
Wilrycx et al. (21)	Netherlands	Stepped-wedge RCT	Mental health network (inpatient and outpatient services)	Combined clinical and non-clinical staff	*N =* 210	Staff: Knowledge	Recovery Knowledge Inventory (RKI; Bedregal et al. (27))	Some concerns

* *Only outcomes examined in the current review study are listed*.

### Characteristics of Recovery Training

A table providing a summary of the training program characteristics, according to the adapted scorecard domains is presented in Table 2. The scorecard was based on the following evidence-based design and delivery techniques identified by Bluestone et al. (16): (1). A delivery method that prioritises learner engagement and stimulation, rather relying on passive transfer of knowledge (*Learner engagement*); (2). Provision of targeted and individualised feedback to the learner (*Feedback*) (3). Ongoing exposure to material, through repeated training or follow-up sessions (*Frequency*), and (4). *In-vivo*, or clinically integrated, teaching to allow for practise within the work environment (*Setting*).

**Table 2:** Training design and delivery characteristics of the included studies [scorecard adapted from (16)].

**Study**	**Learner engagement (Passive vs. interactive)**	**Feedback (Provided vs. not provided)**	**Frequency (Delivered once vs. repeated delivery/follow-up)**	**Setting (Classroom vs. *in-vivo*)**	**Score**
Bitter et al. (19)	Interactive Delivered in person; Direct instruction by trainers; Sessions are participatory, including both theory and “on-the-job” training	Feedback Provided as part of “training on-the-job” and coaching sessions	Repeated Seven sessions; follow-up coaching provided after training completed	Both Combination of “theory meetings” and “training on-the-job”	**4/4**
Bitter et al. (20)	As above	As above	As above	As above	**4/4**
Mak et al. (22)	Interactive Delivered in person, with a single video presentation; Combination of didactic teaching, interactive games, discussion, quiz; Includes service user/carer presentation	Unclear Unclear if feedback provided as part of training	Delivered once Two, three-hour sessions; delivered once; no follow-up sessions	Classroom-based Classroom-based psychoeducation; no *in-vivo* training provided	**1/4**
Meadows et al. (23)	Interactive Delivered in-person, by various trainers, including clinicians and “consumer academics”; Coaching sessions were experiential in nature; Training described as “Active learning sessions”	Feedback Provided as part of coaching	Unclear Unclear if follow-up telephone support and booster sessions included (as provided in REFOCUS trial)	Unclear Unclear if *in-vivo* element included	**2/4**
Pollard et al. (24)	Interactive Delivered in person, with single video presentation; Combination of lectures, group discussions, service user/carer presentations and site visits	Unclear Unclear if feedback is provided as part of training	Delivered once Six sessions and six site visits; delivered once; no follow-up sessions	Classroom-based Includes community visits, but no active, *in-vivo* training provided	**1/4**
Slade et al. (5)	Interactive Delivered in-person, by various trainers, including professionals and ex-service users; Participatory training sessions; Reflective group sessions	Feedback Provided as part of direct instruction and reflective group sessions	Repeated Multiple sessions, with follow-up telephone support; Booster sessions; Reflective group session; Structured supervision	Unclear Unclear if *in-vivo* element included	**3/4**
Wilrycx et al. (21)	Interactive Delivered in person, by expert by experience, and rehabilitation professional; Highly interactive; Group discussions; Group exercises; Homework assigned	Unclear Unclear if feedback is provided as part of training	Delivered once Two, two-day sessions; delivered once; no follow-up sessions	Classroom-based Classroom-based; no *in-vivo* training provided	**1/4**

#### Aims of the Training Programmes

Although all training programs self-identified as “recovery” interventions, or comprised of recovery-oriented components, the stated aims varied. These were largely split between those that aimed to improve outcomes for service users, and those that aimed to change the beliefs/attitudes/behaviours of staff. The CARe methodology aimed to “*to support a client in his/her recovery and to improve his/her quality of life, (through) realising goals and wishes; handling vulnerability; and improving the quality of the client's social environment*” [(19), p. 2]. Both the REFOCUS (5) and the REFOCUS-PULSAR interventions aimed to “*promote recovery through changes in the skills, knowledge, behaviour, and values of staff and their relationships with consumers*” [(19), p. 104]. The intervention assessed by Wilrycx et al. (21) aimed to “*to create and promote a new culture toward recovery from serious mental illness*” by changing treatment and relationships between providers and service users. The program assessed by Mak et al. (22) aimed to “*promote recovery knowledge and attitudes*” amongst mental health staff, while Pollard et al. (24) examined an intervention designed to improve “*staff attitudes and knowledge regarding psychiatric rehabilitation and recovery*.”

#### Duration/Frequency

The examined recovery training programs varied in terms of duration and frequency. The psychoeducation program assessed by Mak et al. (22) consisted of 2, 3-h sessions (6 h total). The remaining programs ranged from two, 2-day sessions (~16 h total) (21) to seven meetings, including three full-day theory meetings and four half-day “training on-the job” sessions (~40 h total), and follow-up coaching sessions every 4–6 weeks (20).

#### Model of Delivery

All training interventions utilised a combination of didactic methods and experiential learning approaches, including classroom-based lectures, workshops, quizzes, supervision/coaching sessions, community visits, structured dialogue with service users, and feedback. The majority of interventions had training content delivered, in part, by service users/carers.

### Content and Learning Outcomes

All reviewed training programs contained content that reflected the five domains of the CHIME model (15); Connectedness, Hope, Identity, Meaning, and Empowerment. Though varying in their focus, all training programs, either directly or indirectly, sought to effect change across one or more of the following five areas; recovery-related knowledge, staff attitudes toward recovery, staff skills, recovery-oriented behaviour, and staff-service user relationships. See Table 3 for content summaries for the included studies.

**Table 3:** Training content of the included studies^*^.

**Study**	**Recovery knowledge**	**Attitudes toward recovery**	**Staff skills**	**Recovery-supporting behaviours**	**Staff-service user relationship**
Bitter et al. (19)	Theoretical principles of the CARe methodology (Recovery; Presence; Strengths-orientation Social participation; Resources)	*No explicit emphasis on modifying attitudes*	Explicit instruction in the CARe methodology (Relationship building; Strengths assessment; Goal identification; The “recovery worksheet”; Supporting goal attainment)	Explicit instruction in the CARe methodology (Relationship building; Strengths assessment; Goal identification; The “recovery worksheet”; Supporting goal attainment)	Partnership building Importance of the support relationship Safety and equality within the support relationship Importance of frequent contact
Bitter et al. (20)	As above	As above	As above	As above	As above
Mak et al. (22)	Aspects of recovery “Medical and rehabilitation models of recovery” vs. “consumer-oriented recovery” Best practise Challenges to the implementation of recovery-oriented care	*No explicit emphasis on modifying attitudes*	*No explicit emphasis on skill development*	How to apply recovery “elements” in various scenarios	The relationship, and the role of carers, family members, and support staff
Meadows et al. (23)	Recovery-related knowledge (Meaning; Clinical vs. personal recovery; Stigma etc)	Recovery supporting beliefs and values Identity beyond illness Use of pro-recovery language	Coaching skills Care planning Identify and utilise patient strengths and available resources “Life maps”	Importance of patient preferences in care planning Supporting patient goals Empowering patients	Emphasis on recovery-promoting relationships Understanding patient values “Coaching for recovery”
Pollard et al. (24)	Understanding client-centred and strengths-based approaches Evidence-based rehabilitation practises Awareness of community services The “recovery mission”	Increase hope Belief in patient autonomy Sensitivity to service user/carer experience	Strategies for increasing motivation	Inclusion of consumers and families at all stages	“Listening to the consumer” as a strategy Avoiding paternalism
Slade et al. (5)	Recovery-related knowledge (meaning; clinical vs. personal recovery; stigma etc)	Recovery supporting beliefs and values Identity beyond illness Use of pro-recovery language	Coaching skills Care planning Identify and utilise patient strengths and available resources “Life maps”	Importance of patient preferences in care planning Supporting patient goals Empowering patients	Emphasis on recovery-promoting relationships Understanding patient values “Coaching for recovery”
Wilrycx et al. (21)	Treatment, rehabilitation, and recovery Recovery processes Barriers to recovery Characteristics of recovery support	Beliefs about recovery The importance of service user autonomy and empowerment Workers reflecting on their own recovery processes	Methods to “stimulate and facilitate recovery within the client” Contributing to, rather than directing, client's journey	How to apply principles to practise Professional as a support for the client's “own storey”	Professional as a support for the client's “own storey”

* *These are examples only, and may not provide a complete summary of all training components*.

All assessed programs included an emphasis on improving staff understandings of recovery, recovery principles, and the fundamentals of recovery-oriented support. Some studies explicitly sought to influence staff attitudes, beliefs, and values; targets included pro-recovery values (5, 23), beliefs about recovery (21), hope, and belief in “patient” autonomy (24). In others, the intention of transforming staff attitudes was implied through the training content [e.g., (19, 20)]; for example, presentations by carers and service users about their recovery journey modelled service user/carer involvement, a key component of recovery orientated practise (22). The focus on skill-development varied across studies. Mak et al. (22) evaluated a psychoeducation intervention, thus skill-development was not an emphasis. The remaining studies aimed to develop staff skills in relation to specific support interventions, such as assessing service users' strengths, life mapping and care planning (5, 19, 20, 23) and the style of engagement with service users, such as coaching and motivation-enhancement (5, 23, 24). Similarly, the relative emphasis on staff behaviour change and recovery promoting behaviours varied across studies; training programs targeted social inclusion, patient preferences and empowerment (5, 23, 24), and provided guidance on how to support goal attainment (19, 20) and apply recovery-principles more generally (21, 22). Reflecting the socio-environmental nature of personal recovery, staff-service user relationships were also a prominent target of the training interventions; all programs addressed this aspect of practise.

### Outcomes

A summary of findings, stratified by outcome, is presented in Table 4.

**Table 4:** Summary of effectiveness data, across outcome variables (group x time effects, unless otherwise indicated).

	**Service user:** ** Self-reported recovery**	**Staff:** ** Recovery knowledge**	**Staff:** ** Recovery practice**	**Service user:** ** Symptoms**	**Service user:** ** Functioning**
Bitter et al. (19)	Not significant χ^2^ = 1.28; *p =* 0.53	–	–	–	Not significant χ^2^ = 4.64; *p =* 0.10
Bitter et al. (20)	–	Not significantχ^2^ = 4.19; *p =* 0.12	–	–	–
Mak et al. (22)	–	**Significant***F =* 35.19; *p < * 0.001	–	–	–
Meadows et al. (23)	**Significant (S1)** ADif = 3.7; *p =* 0.023	–	Not significant ADif = 2.0; *p =* 0.65	–	Not significant ADif = 0.9; *p =* 0.80
Pollard et al. (24)	–	**Significant***F =* 25.7; *p < * 0.001	–	–	–
Slade et al. (5)	Not significant *b* = 0.63; *p =* 0.55	Not significantχ^2^ = 2.95; *p =* 0.23	Not significant *b* = −2.43; *p =* 0.41	Not significant*b* = 1.85; *p =* 0.15	**Significant** *b* = 5.90; *p < * 0.0001
Wilrycx et al. (21)	–	Not significantχ^2^ = 1.64; *p =* 0.65	–	–	–

#### Service-User Self-Reported Recovery

Of the seven included papers, three examined service-user, self-reported recovery as an outcome of the intervention. Results were mixed: one of the three trials reported positive results. Bitter et al. (19) assessed change in personal recovery, as measured by the Mental Health Recovery Measure [MHRM; (25)], at baseline and 10- and 20-months post intervention. Using a linear mixed modelling approach, no significant time by intervention effect was identified (*X*^2^ = 1.28; *p* = 0.53). This non-significant finding was replicated when the model controlled for age, gender, having a partner, symptoms, amount of support, recovery-promoting relationship, and recovery knowledge of the professionals. In the REFOCUS trial, Slade et al. (5) used the Questionnaire about the Process of Recovery [QPR; (28)] to examine change in personal recovery. Between baseline and 1-year follow-up, analysis showed no significant effect of REFOCUS on overall recovery (total QPR score): *b* = 0.63 (*p* = 0.55; 95%C = −1.41 to 2.67). However, the REFOCUS-PULASR trial (23) demonstrated a significant effect on QPR scores (ADif = 3.7; *p* = 0.023).

#### Staff Recovery Knowledge

Staff recovery knowledge was the most commonly assessed outcome, with five of seven papers examining changes in this variable over time. As with personal recovery, the findings were mixed. Non-significant effects were reported by Bitter et al. (20) (χ^2^ = 4.19; *p* = 0.12), Slade et al. (5) (χ^2^ = 2.95; *p* = 0·23), and Wilrycx et al. (21) (χ^2^ = 1.64; *p* = 0.65), over follow-up periods ranging from 6 to 20 months. Both Mak et al. (22) and Pollard et al. (24) demonstrated a significant improvement in staff recovery knowledge, using the Recovery Knowledge Inventory (RKI; *F* = 35.19; *p* < 0.001) and the Beliefs, Goals, and Practises in Psychiatric Rehabilitation Questionnaire (PBGPPR; *F* = 25.7; *p* < 0.001), respectively. It must be noted, however, that, in both studies, this staff recovery knowledge was assessed before and immediately after the training, thus the long-term stability of these changes was not examined.

#### Staff Recovery Practise

Two of the included papers considered the effect of the training intervention on staff recovery practise. Meadows et al. (23) used the Importance of Services in Recovery questionnaire [INSPIRE; (32)], while Slade et al. (5) used the Recovery Practise Scale, a non-standardised instrument, to measure practise change over time. Neither study demonstrated a significant effect of the training intervention on staff recovery practise (ADif = 2.0; *p* = 0.65) (*b* = −2.43; *p* = 0.41).

#### Service User Symptoms

Only one study examined the effect of recovery training on service user symptoms over time. Slade et al. (5) found no significant effect on this variable between baseline and 1-year follow-up; *b* = 1.85; *p* = 0.15.

#### Service User Functioning

Of the seven included papers, three considered service user functioning as an outcome variable. As with all previously reported findings, the results were mixed. Using the Social Functioning Scale (SFS), Bitter et al. (19) found no significant difference in service user functioning between baseline and 20-month follow up; χ^2^ = 4.64; *p* = 0.10. Similarly, Meadows et al. (23) found no change in Global Assessment of Functioning (GAF); ADif = 0.9; *p* = 0.80. Conversely, in the REFOCUS trial, Slade et al. (5) demonstrated a significant effect of training on service user GAF scores over time, *b* = 5.90 (*p* < 0.0001; 95%C = 2.61–9.18).

### Associations Between Training Characteristics, Content, and Efficacy

The synthesised findings showed no clear relationship between training design and delivery characteristics, as measured by the adapted scorecard, training content, and reported outcomes.

## Discussion

### Summary of Findings

This systematic review aimed to describe and compare the characteristics and efficacy of existing recovery training packages for mental health staff. The included papers showed variation in the aims, frequency and duration of the training interventions evaluated although they all included content consistent with the five-domains of the CHIME model (15). Design and delivery characteristics of the training programs were evaluated using an adapted scorecard, reflecting evidence-based training methods (16). All studies used direct, in-person teaching, and prioritised interactive, experiential learning, however a number were limited by the absence of feedback, the use of one-off, rather than repeated/follow-up sessions, and a reliance on classroom-based, rather than *in-vivo*, training.

The efficacy of the interventions was inconsistent. Only three studies examined service user recovery as an outcome, and, of those that did, only one (23) demonstrated a significant effect. Of the five studies that examined staff recovery knowledge as an outcome, only two demonstrated a significant effect; however, for both, this was assessed using a simple pre-post analysis, and provides limited evidence of longer-term maintenance. There was no clear association between the delivery and design characteristics of the interventions and outcomes. Overall, these data provide limited evidence for the efficacy of recovery-focused training interventions, particularly in relation to service user outcomes. These findings are largely consistent with those of a recent review by Jackson-Blott et al. (13), although, likely due to the inclusion of uncontrolled/non-randomised studies, the authors found more a uniform influence of training on staff knowledge, attitudes and competencies.

### Explanations for Findings

These somewhat disappointing findings are not uncommon in the mental health field; staff training interventions frequently fail to demonstrate an effect on staff behaviours and service user outcomes [e.g., (33)]. In attempting to explain their results, authors posited a range of potential explanations, including staff factors (readiness to change, age, existing use of recovery-orientation), service-user factors (degree of illness acuity), structural factors (budget cuts, and service reorganisation), and study-design factors (outcome measure sensitivity and relative brevity of follow-up period).

An explanation that was consistently identified in the reviewed studies related to challenges around implementation. Staff turnover, low morale, poor leadership, and limited “buy-in” were linked to poor fidelity and outcomes. The role of implementation in influencing outcomes was perhaps best demonstrated by the *post-ho*c analyses in the REFOCUS study (5). When a distinction was made between high participation teams, low participation teams and controls (based on attendance and engagement with the training), analyses demonstrated that high staff participation, but not low staff participation, was associated with higher service user self-reported recovery and staff recovery practise. Implementation science has produced a vast array of models describing factors that should be considered when embedding new ways of working [e.g., (34–36)]; it is beyond the scope of the current review to examine these in detail, however it is important to emphasise that staff, organisational and ecological analyses, identifying barriers and facilitators of change, prior to the implementation of new ways of working (such as recovery training), are essential. An a priori examination of potential implementation issues may allow researchers to identify, and manage, potential issues prior to the commencement of training-focused studies, thus ensuring more consistent outcomes. Parallel or nested implementation studies that evaluate implementation outcomes, such as acceptability or appropriateness, are also recommended (see (37) [REFOCUS]). Separating intervention outcomes from implementation outcomes provides additional insights regarding non-significant findings, and can assist in distinguishing between an ineffective intervention, and an effective intervention that had been implemented unsuccessfully (38).

Another important, though less frequently discussed, reason for our findings is the possibility that the training interventions themselves cannot address broader factors that may influence service user recovery. Typically, the change model for recovery training interventions suggests that by improving staff practise, service user experience is enhanced, which in turn supports service user recovery. However, this model is likely too simplistic, and overlooks the fact that staff practise may, in fact have a limited impact on the recovery processes of individual service users. Various social determinants of health, commonly experienced by individuals with SMI, such as unemployment, poverty, isolation, and stigma (39) are likely to have an important impact on service user recovery, beyond staff practise. Indeed, common critiques of the recovery concept focus on the individualistic/neo-liberal conceptualisation of personal recovery, and its inadequacy in addressing the social, cultural, and systematic obstacles faced by marginalised groups [see (40, 41)].

### Recovery Training for UK Supported Accommodation

With a growing evidence-base highlighting the association between recovery-oriented practise and service-user outcomes, a pressing questions remains: How do we equip the supported accommodation workforce to deliver this form of support? Training interventions that aim to develop the skills, knowledge, behaviour, and values of staff appear to be a logical approach to addressing this issue, however the findings of the current review highlight the significant challenges currently faced. To maximise the potential benefit of any recovery training developed for use within this sector, attention must be paid to content, characteristics and delivery of the intervention, alongside a detailed consideration of the context and composition of these services.

#### Considerations: Training Content, Characteristics, and Delivery

Although debate still exists regarding the exact nature of personal recovery, how it to be understood, how it should be reflected in service design, and how recovery-processes can be supported by staff, carers, and families, there is a striking consistency in the literature regarding its central tenets (see (42) for a recent review). The CHIME model (15), emphasising concepts of connectedness, hope, identity, meaning and empowerment is widely endorsed, and, reassuringly, all training programs included in this review include content that reflect the model. However, as described, the training programs did not consistently lead to desired outcomes, suggesting that the inclusion of these recovery-specific elements may not be sufficient to generate benefits for service users. This observation must be considered when developing any recovery training intervention for use in supported accommodation settings; looking beyond the content, to consider the characteristics and delivery methods of an intervention, is imperative.

In a recent integrative review of 37 systematic reviews and 32 RCTS, Bluestone et al. (16) identified a range of evidence-based training approaches that supported the development of knowledge, skills and practise of health staff. *In-vivo* approaches and learner feedback were found to be effective, while passive methods, such as lectures and self-directed reading, had little impact on outcomes. Interventions that were repeated, rather than delivered within a single session, were more effective. These findings are similar to those reported by Lyon et al. (43), however, the authors observe that it is “*unlikely that the use of traditional workshop models or any single strategy will result in success*” (p. 248). Indeed, combining delivery approaches appears to have a cumulative impact on the effectiveness of staff training. Although design and delivery characteristics of the training programs included in this review did not appear to be associated with outcomes, the most commonly neglected (or unreported) training components were provision of feedback to learners, repeated delivery/follow-up sessions and *in-vivo* training. Researchers should ensure that future interventions attempt to include these elements, and may consider the inclusion of additional behavioural components shown to improve the quality of care in health settings, such as regular supervision (44). In the absence of strong efficacy data, researchers must rely on the broader evidence to inform the design and delivery of new approaches.

#### Considerations: Resources

The impact of austerity and budget cut-backs on mental health services has been well-documented (45, 46). The supported accommodation sector in the UK has not been immune to this shift; recent data has highlighted a progressive reduction in funding for support costs in supported accommodation over time (47). Budget restrictions inevitably impact how services are delivered and the development opportunities made available for staff. In a recent report on skills, training and employability issues in the mental health sector, “*limited budget for training*” and “*limited time for training*” were cited by providers as the primary reasons for skills deficits amongst staff (48). As stated by a provider: “*with reduced funding, zero hour contracts and lack of staff time it is becoming a luxury to provide anything more than mandatory training*” [(49), p. 18].

These resource constraints must be taken into account when considering recovery training for supported accommodation staff. The majority of the training approaches reviewed in this paper include multiple group sessions and follow-up support; it is unclear whether these time and cost burdens could be tolerated by supported accommodation providers. As noted above, however, for training to be effective, extended/repeated contact must be prioritised (16). In developing recovery training for this sector, researchers must manage the tension between resource limitations and evidence-based practise; creative approaches to training delivery, utilising low-cost methods, such as peer-coaching or computer-delivered interventions, could be considered (50).

#### Considerations: Workforce

A recent systematic review of the views of mental health staff highlighted the persistence of symptom-focused and biomedical conceptualisations of recovery amongst clinically trained staff (51). Due to their non-clinical background, supported accommodation staff may, therefore, be uniquely positioned to deliver recovery-oriented care; indeed, supported accommodation services in the UK demonstrate higher recovery-based practise scores when compared to inpatient rehabilitation units (as measured by the Quality Indicator for Rehabilitative Care) (52, 53). Support provision within supported accommodation settings is typically psychosocially-focused (54), with clinical tasks, such as medication management and symptom monitoring, managed by statutory services; thus, the core remit of these services shares a natural overlap with recovery-supporting practise.

Despite these potential strengths, a number of workforce issues, pertinent to the supported accommodation sector, should also be considered. Staff turnover in these settings is a significant problem. Though accurate and precise data is difficult to access, trends in the broader adult social care sector are likely applicable to supported accommodation settings. Recent statistics suggest that the staff turnover rate in the English adult social care sector was 30.8% in 2018/19, equating to 440,000 people leaving their jobs (55), with younger workers and those paid less more likely to leave their role. Turnover represents a challenge for providers, whereby training may be viewed as a “waste” if staff are deemed likely to leave. Training packages must therefore be flexible and designed in such a way that new staff are able to access evidence-based materials quickly, in order to develop or enhance relevant skills.

Research repeatedly highlights that many “soft skills” that are essential to recovery-oriented practise, such as empathy and effective listening, are considerably more difficult to teach than “hard skills,” such as care planning (48). The centrality of these “soft skills” to recovery-based practise suggests that recruitment of appropriate candidates, who already possess many of these proficiencies, rather than using training to embed or develop these skills, may be an appropriate way to ensure that supported accommodation staff have the prerequisite attitudes and values to deliver recovery-oriented support. These observations also have implications for training development. Although attitude change was a common intervention target amongst the reviewed papers, it is possible that training staff in simplified, task, or skill-focused interventions that can support service user recovery, such as shared decision making (56), may be more beneficial for service user outcomes than explicit attempts to modify values. This is particularly relevant in a sector where brief training interventions will likely be necessary, due to the resource limitations described above.

### Recovery Training for Supported Accommodation: Future Research

With the continued emphasis on personal recovery, supported accommodation services have an obligation to deliver evidence-based, recovery-oriented support. As described, however, the sector is facing significant pressures which directly impact its ability to deliver high-quality services; financial restrictions, increasing service-user demand, and high turnover and poor remuneration for staff are some of the difficulties currently faced. A challenge for researchers, therefore, is to develop recovery training interventions that are evaluable and take account of these barriers, whilst still maintaining a focus on comprehensiveness, rigour, and feasibility. One resource-conscious approach may be to build on the strengths of the workforce, aiming to optimise existing, psychosocially focused practises, rather than attempting to introduce new ways of working. As reported above, however, the success of such an intervention will likely depend on the effective integration of a range of evidence-based design and delivery methods.

It must be acknowledged however, that a singular emphasis on recovery training in these settings is unlikely to lead to the desired outcome of improved service user recovery. There is a wealth of literature demonstrating that staff training alone does not consistently result in improved practise (57); essential elements, beyond staff training, include a context that supports the desired behaviour change, visible organisational support, active and engaged leadership, and the redesign of workflows to “*build new practise into the fabric of daily work*” [(58), p. 361]. Indeed, some of the more robust training packages reviewed here have attempted to address these factors by including multi-level interventions that target frontline staff, supervision procedures and team culture; this approach reflects best-practise, and should be a consideration for any intervention developed for supported accommodation services. More broadly, the sector may consider adopting Quality Improvement (QI) methods to improve practise and service user outcomes. QI takes a systematic and data-driven approach to “problem-solving”; solutions to problems are identified, tested and implemented at a local level, with an awareness of the complexities of the immediate environment (59). These methods are becoming more common in statutory mental health services in the UK (60), and could be incorporated into, or delivered alongside, novel recovery training programmes within the supported accommodation sector.

Ultimately, in order to support service user recovery, staff behaviour change should be seen as a component of the broader goal of service transformation, rather than the sole driver; training for the supported accommodation workforce will be an essential element, but must be part of an array of interventions designed to support the full spectrum of recovery needs of individuals. A large body of research has highlighted factors that may influence service user recovery in supported accommodation settings, beyond staff competencies, such as the physical design and restrictiveness of the environment, level of integration with other mental health services, privacy, security, service-user relationships and loneliness (61–63). These elements may represent meaningful targets when undertaking a service redesign that aims to improve recovery outcomes for service users. Although quantitative evaluations of service reorganisations are uncommon, a number of case studies and service models exist that can guide and support providers in enacting such systemic change [e.g., (64)].

## Strengths and Limitations

The current review has a number of strengths. We developed a thorough search strategy, including only randomised designs, and applied it to a large number of databases; these decisions increase confidence in the comprehensiveness of the search itself, and the quality of the included studies. We used an adapted scorecard, based on current quantitative evidence, to assess the design and delivery characteristics of the included interventions, allowing us to identify elements of the training programs that may have impacted their efficacy. To avoid definitional issues, we opted to include only studies that evaluated interventions that were identified by the authors as “recovery” training. It is possible that by using this approach, we may have overlooked relevant training interventions that did not use the term “recovery” to describe the nature or focus of the programme. Relatedly, due to this decision, the current review did not include training interventions that target specific aspects of personal recovery (for example, social inclusion [Connectedness]). In interpreting the findings of the review, it is important to highlight that none of the included studies delivered training to an exclusively non-clinical staff group; as supported accommodation services in the UK are staffed by non-clinical staff, data from the included studies may not be fully generalisable to these settings. in addition, although we assume that many of the observations reported above (particularly relating to resource and workforce issues) will be applicable to supported accommodation services internationally, it must be acknowledged that housing models and approaches to service organisation vary between countries; the conclusions reported here were drawn specifically relation to the UK supported accommodation context. Finally, although we are confident in the thoroughness of the search strategy, the strategy itself was developed without input from a specialist librarian and may not be fully comprehensive.

## Conclusion

Currently, the evidence-base supporting recovery training for mental health staff is underdeveloped and inconsistent. The current review, examining the characteristics and effectiveness of these interventions, found limited evidence to suggest an impact on service user and staff outcomes; no clear conclusions can be drawn from the available data. This highlights a clear gulf between aspirations of embedding the concept of recovery within mental health policies and the realities of operationalising recovery in training and staff practise; recovery-oriented services, as a goal of mental health systems, is commendable, however these findings raise questions regarding how we best equip the workforce to deliver this form of support.

In considering the development of recovery training materials for supported accommodation staff, little guidance can be taken from the reviewed literature; as described, there is no clear association between recovery training content, duration and delivery method, and outcomes. Any training, therefore, must be informed by learning theories and evidence relating to training effectiveness taken from other fields, and developed with consideration to the unique contextual and organisational characteristics of these services, and of the individuals, they support.

## Data Availability Statement

The original contributions presented in the study are included in the article/Supplementary Material, further inquiries can be directed to the corresponding author/s.

## Author Contributions

PM, HK, and BL-E designed the study. PM undertook searches, extracted and analysed the data, and drafted the manuscript. CD-L assisted with screening and critically commented on draughts of the manuscript. HK and BL-E supervised the study, contributed to interpretation of the data, and critically commented on draughts of the manuscript. All authors read and approved the final manuscript.

## Conflict of Interest

The authors declare that the research was conducted in the absence of any commercial or financial relationships that could be construed as a potential conflict of interest.
