# Supplementary material for: A Systematic Review of the Characteristics and Efficacy of Recovery Training for Mental Health Staff: Implications for Supported Accommodation Services
Source: Front Psychiatry. 2021 May 14;12:624081. doi: 10.3389/fpsyt.2021.624081 (PMC8160251; doi:10.3389/fpsyt.2021.624081)
Supplement: Supplementary file 1 [file Table_1.DOCX]

Search strategy

| **MEDLINE (OVID)** | | **EMBASE (OVID)** | | **PsychInfo (OVID)** | |
| --- | --- | --- | --- | --- | --- |
|  | *Terms* |  | *Terms* |  | *Terms* |
| 1 | exp Mental Disorders/ | 1 | exp Mental Disorders/ | 1 | exp Mental Disorders/ |
| 2 | Mental Health/ | 2 | exp Mental Health/ | 2 | Mental Health/ |
| 3 | ((psychol* or psychiat* or mental*) adj (illness* or disorder* or problem* or disease* or disab*)).tw. | 3 | ((psychol* or psychiat* or mental*) adj (illness* or disorder* or problem* or disease* or disab*)).tw. | 3 | ((psychol* or psychiat* or mental*) adj (illness* or disorder* or problem* or disease* or disab*)).tw. |
| *4* | *1 or 2 or 3* | *4* | *1 or 2 or 3* | *4* | *1 or 2 or 3* |
| 5 | (staff or worker$ or care coordinator$ or personnel or employee$ or clinician$ or professional$ or practitioner$ or provider$ or physician$ or psychiatrist$ or doctor$ or nurse$ or occupational therapist$ or social worker$ or psychologist$).tw. | 5 | (staff or worker$ or care coordinator$ or personnel or employee$ or clinician$ or professional$ or practitioner$ or provider$ or physician$ or psychiatrist$ or doctor$ or nurse$ or occupational therapist$ or social worker$ or psychologist$).tw. | 5 | (staff or worker$ or care coordinator$ or personnel or employee$ or clinician$ or professional$ or practitioner$ or provider$ or physician$ or psychiatrist$ or doctor$ or nurse$ or occupational therapist$ or social worker$ or psychologist$).tw. |
| 6 | exp education, medical/ or exp education, nursing/ or exp education, continuing/ or exp inservice training/ | 6 | exp education, medical/ or exp education, nursing/ or exp education, continuing/ or exp inservice training/ | 6 | exp PERSONNEL TRAINING/ or exp PSYCHIATRIC TRAINING/ or exp MENTAL HEALTH INSERVICE TRAINING/ |
| 7 | (training or education or teaching or skill$ or skill development or workshop$ or professional development or instruction).tw. | 7 | (training or education or teaching or skill$ or skill development or workshop$ or professional development or instruction).tw. | 7 | (training or education or teaching or skill$ or skill development or workshop$ or professional development or instruction).tw. |
| 8 | *6 or 7* | 8 | *6 or 7* | 8 | 6 or 7 |
| 9 | exp Mental Health Recovery/ | 9 | exp Mental Health Recovery/ | 9 | exp "RECOVERY (DISORDERS)"/ |
| 10 | (recovery or personal recovery or social recovery or mental health recovery).tw. | 10 | (recovery or personal recovery or social recovery or mental health recovery).tw. | 10 | (recovery or personal recovery or social recovery or mental health recovery).tw. |
| 11 | *9 or 10* | *11* | *9 or 10* | 11 | *9 or 10* |
| 12 | Randomized Controlled Trial.pt. |  |  |  |  |
| 13 | (random$ or placebo$ or single blind$ or double blind$ or cluster).tw. | 12 | (random$ or placebo$ or single blind$ or double blind$ or cluster).tw. | 12 | (random$ or placebo$ or single blind$ or double blind$ or cluster).tw. |
| 14 | *12 or 13* |  |  |  |  |
| **15** | **4 and 5 and 8 and 11 and 14** | **13** | **4 and 5 and 8 and 11 and 12** | **13** | **4 and 5 and 8 and 11 and 12** |
| **16** | **Limit to: yr="1990 -Current"** | **14** | **Limit to: yr="199 0 -Current"** | **14** | **Limit to: yr="1990 -Current"** |

| **Cochrane Library** | |  | **CINAHL Plus (EBSCO)** |  | **Web of Science** |
| --- | --- | --- | --- | --- | --- |
| *Terms* | |  | *Terms* |  | *Terms* |
| 1 | MeSH descriptor: [Mental Disorders] explode all trees | S1 | (MH "Mental Disorders+") | 1 | TS=("mental illness" or "mental health") |
| 2 | (“Mental Health”):ti,ab,kw | S2 | (MH "Mental Health") | 2 | TS=((psychol* or psychiat* or mental*) NEAR/0 (illness* or disorder* or problem* or disease* or disab*)) |
| 3 | (psychol* or psychiat* or mental*) next (illness* or disorder* or problem* or disease* or disab*):ti,ab,kw | S3 | TI (psychol* or psychiatr* or mental*) W0 (illness* or disorder* or problem* or disease* or disab*) OR AB (psychol* or psychiatr* or mental*) W0 (illness* or disorder* or problem* or disease* or disab*) |  |  |
| 4 | #1 or #2 or #3 | *S4* | *S1 OR S2 OR S3* | 3 | #1 or #2 |
| 5 | (staff or worker* or “care coordinator*” or personnel or employee* or clinician* or professional* or practitioner* or provider* or physician* or psychiatrist* or doctor* or nurse* or “occupational therapist*” or “social worker*” or psychologist*):ti,ab,kw | S5 | TI (staff or worker* or “care coordinator*” or personnel or employee* or clinician* or professional* or practitioner* or provider* or physician* or psychiatrist* or doctor* or nurse* or “occupational therapist*” or “social worker*” or psychologist*) OR AB (staff or worker* or “care coordinator*” or personnel or employee* or clinician* or professional* or practitioner* or provider* or physician* or psychiatrist* or doctor* or nurse* or “occupational therapist*” or “social worker*” or psychologist*) | *4* | TS=(staff or worker* or “care coordinator*” or personnel or employee* or clinician* or professional* or practitioner* or provider* or physician* or psychiatrist* or doctor* or nurse* or “occupational therapist*” or “social worker*” or psychologist*) |
| 6 | MeSH descriptor: [Education, Medical] explode all trees | S6 | (MH "Education, Medical+") OR (MH "Education, Nursing+") OR (MH "Education, Allied Health") OR (MH "Education, Social Work") OR (MH "Education, Occupational Therapy") OR (MH "Education, Continuing+") |  |  |
| 7 | MeSH descriptor: [Education, Nursing] explode all trees |  |  |  |  |
| 8 | MeSH descriptor: [Education, Nonprofessional] this term only |  |  |  |  |
| 9 | MeSH descriptor: [Education, Continuing] explode all trees |  |  |  |  |
| 10 | MeSH descriptor: [Inservice Training] explode all trees |  |  |  |  |
| *11* | (training or education or teaching or skill* or “skill development” or workshop* or “professional development” or instruction):ti,ab,kw | S7 | TI (training or education or teaching or skill* or “skill development” or workshop* or “professional development” or instruction) OR AB (training or education or teaching or skill* or “skill development” or workshop* or “professional development” or instruction) | 5 | TS=(training or education or teaching or skill* or "skill development" or workshop* or "professional development" or instruction) |
| 12 | *#6 or #7 or #8 or #9 or #10 or #11* | *S8* | *S6 or S7* |  |  |
| 13 | MeSH descriptor: [Mental Health Recovery] explode all trees | S9 | (MH "Recovery") |  |  |
| 14 | (recovery or “personal recovery” or “social recovery” or “mental health recovery”):ti,ab,kw | S10 | TI (recovery or “personal recovery” or “social recovery” or “mental health recovery”) OR AB (recovery or “personal recovery” or “social recovery” or “mental health recovery”) | 6 | TS=(recovery or "personal recovery" or "social recovery" or "mental health recovery") |
| 15 | #13 or #14 | S11 | *S9 or S10* |  |  |
| 16 | MeSH descriptor: [Randomized Controlled Trials as Topic] explode all trees | S12 | (MH "Randomized Controlled Trials+") |  |  |
| 17 | (random* or placebo* or “single blind*” or “double blind*” or cluster):ti,ab,kw | S13 | TI (random* or placebo* or “single blind*” or “double blind*” or cluster) OR AB (random* or placebo* or “single blind*” or “double blind*” or cluster) | 7 | TS=("random* controlled trial" or "placebo*" or "single blind*" or "double blind*" or cluster) |
| 18 | #16 or #17 | **S14** | S12 or S13 |  |  |
| 19 | #4 and #5 and #12 and #15 and #18 | **S15** | **S4 and S5 and S8 and S11 and S14** | *8* | #7 AND #6 AND #5 AND #4 AND #3 |
| 20 | **Limit to: Publication Year from 1990 to 2019, in Trials** | **S16** | **Limit to: Published Date: 19900101-20190231** | 9 | *Unable to apply limits* |
